# Evaluation of Dentin-Pulp Complex Response after Conservative Clinical Procedures in Primary Teeth

**DOI:** 10.5005/jp-journals-10005-1509

**Published:** 2018-06-01

**Authors:** Bianca Mello, Tassia C Stafuzza, Luciana Vitor, Daniela Rios, Thiago Silva, Maria Machado, Thais M Oliveira

**Affiliations:** 1 PhD Student, Department of Pediatric Dentistry, Bauru School of Dentistry University of Sao Paulo, Sao Paulo, Brazil; 2 PhD Student, Department of Pediatric Dentistry, Bauru School of Dentistry University of Sao Paulo, Sao Paulo, Brazil; 3 PhD Student, Department of Pediatric Dentistry, Bauru School of Dentistry University of Sao Paulo, Sao Paulo, Brazil; 4 Professor, Department of Pediatric Dentistry, Bauru School of Dentistry University of Sao Paulo, Sao Paulo, Brazil; 5 Professor, Department of Pediatric Dentistry, Bauru School of Dentistry University of Sao Paulo, Sao Paulo, Brazil; 6 Professor, Department of Pediatric Dentistry, Bauru School of Dentistry University of Sao Paulo, Sao Paulo, Brazil; 7 Associate Professor, Department of Pediatric Dentistry, Bauru School of Dentistry University of Sao Paulo, Sao Paulo, Brazil

**Keywords:** Deciduous, Dental caries, Dental pulp capping, Tooth.

## Abstract

**Introduction:**

Although selective caries tissue removal decreases the number and diversity of bacteria, stops the caries process, and reduces the risk of pulp exposure, the studies on the minimally removal of caries tissue are limited and further clinical research is necessary in this field.

**Aim:**

This study aimed to evaluate through clinical and radiographic assessments the *in vivo* response of the dentin-pulp complex of human deciduous teeth after either partial or total caries removal (TCR).

**Materials and methods:**

A total of 49 deciduous molars of children aged between 5 and 9 years were carefully selected. The teeth were divided into two groups: Group I: Partial removal of caries; group II: Total removal of caries. Clinical and radiographic evaluations were performed during the period of 4 to 6 months after the procedure. The intraexam-iner reproducibility was determined by Kappa test. Fisher’s exact test was used to determine the statistical difference between groups.

**Results:**

All teeth showed clinical success during the 4- to 6-month evaluation period. The radiographic evaluation showed 94.2 and 89.6% of success rate in groups I and II respectively. Radiographic results did not show statistically significant differences between the studied groups (p > 0.05).

**Conclusion:**

The partial caries removal (PCR) showed satisfactory clinical and radiographic outcomes, suggesting that this minimally invasive approach might replace the TCR when correctly indicated.

**How to cite this article:** Mello B, Stafuzza TC, Vitor L, Rios D, Silva T, Machado M, Oliveira TM. Evaluation of Dentin-Pulp Complex Response after Conservative Clinical Procedures in Primary Teeth. Int J Clin Pediatr Dent 2018;11(3):188-192.

## INTRODUCTION

Currently, the caries lesion treatment approaches are more conservative and less invasive to arrest the progression of carious lesions. The initial carious lesion, treated at correct time, can be arrested, and possibly remineralized. The technological advances allow different alternatives in dentistry to find an option enabling better clinical efficacy without side effects. As caries is a disease with pathologic basis of dental biofilm, it is more important to prevent the appearance of new lesions and control existing lesions than focus primarily on the removal of tissue.^1^ In this context, pediatric dentistry has shifted to minimally invasive treatments that avoid more complex, time-consuming procedures, and the child’s discomfort.

The PCR is based on the principle that careful dentin removal prevents further physical damage to the tooth and reducing the possibility of pulpal exposure.^2,3^ The TCR of deep lesions may result in pulp exposure requiring more invasive treatments.^4^ The PCR is based on the changing of the microenvironment of contaminated dentin underlying the restoration, maintaining tooth structure and pulp vitality.^5^ The PCR maintains pulp vitality through the excavation to leathery or firm dentin at the pulpal wall of the cavity.^6^ For this purpose, PCR involves the removal of infected dentin and preserves affected dentin, which, once sealed by the restorative material, is able to remineralize due to the absence of substrate.^7-9^ It is not easy to determine exactly the amount of tissue to be removed,^6,10-12^ so the subjective tactile sensation is the best guide.^6^ However, the studies on the minimal removal of caries tissue are relatively limited and further research is necessary in this field. The literature lacks scientific evidences for defining a safe and adequate protocol to indicate PCR in primary teeth.^2^

Accordingly, clinical studies comparing PCR with conventional caries removal methods are justified because PCR has exhibited good outcomes and the understanding of these novel carious tissue management techniques is still necessary. So, this study aimed to assess clinically and radiographically the *in vivo* dentin-pulp complex response of human primary teeth after PCR and TCR.

## MATERIALS AND METHODS

This study was submitted and approved by the institutional review board regarding the ethical aspects (protocol number #20816913.5.0000.5417). All parents/legal guardians of the study participants signed an informed consent. This study has been conducted in full accordance with the World Medical Association Declaration of Helsinki.

### Sample Selection

Inclusion criteria comprised children aged between 5 and 9 years, both genders, with a maxillary/mandibular primary molar affected by deep caries (more than 2/3 of carious dentin); without sensitivity and/or spontaneous pain; without pulp exposure; without excessive tooth mobility; without fistula or abscess; without internal root resorption or external root resorption of more than 2/3 of the root on radiograph; without furcal and periapical lesion, and with restorative possibility. Children with systemic diseases; history of allergy to the latex from dental dam; and history of allergy to local anesthetic were excluded from the study. The parents/legal guardians were instructed regarding the procedure and signed the informed consent.

### Sample Size Calculation

The sample size was calculated so that the number of selected children would project the representative estimate to conduct the study. For this purpose, we used the difference of 54% between the experimental and control groups from the previous study of Bressani et al.^13^ The minimum sample size was estimated as 12 teeth per group to detect that difference with a level of significance of 5% and power of 20%. Considering the drop-out rate, the minimum sample size was determined to be 17 teeth.

### Clinical Procedures

The primary teeth were divided into two groups: Group I: PCR and group II: TCR. The clinical and radiographic procedures were carried out by a single operator previously trained and calibrated.

Initially, a periapical radiograph of the tooth selected for the study was taken with the use of film holders. The clinical technique comprised the following steps: topical anesthesia, inferior alveolar nerve anesthesia (mandible), or infiltrative anesthesia (maxilla) with local anesthetic (Articaine 4% with 1:100,000 epinephrine); removal of all unsupported enamel with high-speed burs, TCR from the lateral dentin walls with low-speed round steel or carbide burs (sizes 4, 5, and 6—KG Sorensen® Sao Paulo-BR). In group I (PCR), the infected dentin was removed, while the affected dentin was maintained on the pulpal wall. In group II (TCR), both infected and affected dentin was removed through low-speed carbide burs and hand excavators. All demineralized dentin was removed to hard dentin, leaving no softened dentin.

For all groups, the cavity was cleaned with air-water syringe and dried with cotton pellet. The calcium hydroxide cement (Hydro C®/Dentsply Philadelphia, USA) was chosen as liner material. Then, a definitive restoration was accomplished with resin-modified glass ionomer (Vitremer™, 3M/ESPE Minnesota, USA). After that, another periapical radiograph was performed.

### Clinical and Radiographic Analysis

The treated teeth were clinically and radiographically evaluated at 4 to 6 months to assess the pulp-dentin complex response.^14,15^ All risks relating to radiographic shots were carefully controlled by using lead apron and thyroid collar during the radiographic exposure and ultra high-speed film was used to allow a shorter exposure time. The periapical radiographs were obtained in a standard manner, with the aid of Han-Shin film holders, with focus/ film distance of approximately 20 cm, by using a radiographic device at 70 kV and 10 mA, with exposure time of 0.5 seconds. E to F speed radiographic films, size 1 (Insight, Kodak) were used. The radiographs were developed manually, through time/temperature technique in the developing solution, followed by intermediary washing with running water for 20 seconds, immersion in the fixing solution for 10 minutes, final washing in running water tank for 10 minutes and environmental drying, thus allowing the final image with good image quality.

During all the clinical and radiographic follow-up period, clinical success comprised of teeth with no pain, mobility, sensitivity to percussion, abscess/fistula, and color alteration.^16,17^ The radiographic success was noted as teeth with no internal and external root resorption, furcal/ periapical lesion, and advanced rhizolysis stage.^16,17^ Two examiners, specialists in pediatric dentistry, trained, calibrated, and blinded regarding treatment types, performed the analyses. Intraexaminer agreement evaluated by Kappa test was 0.786. All data were registered on the patient’s chart for posterior analysis.

### Statistical Analysis

Data were analyzed by Statistical Package for the Social Sciences software version 21 (IBM, Armonk, New York, USA). Intraexaminer reproducibility was determined by Kappa test. Fisher’s exact test was used to determine the statistical differences between groups. A level of significance of 5% was adopted.

## RESULTS

One hundred primary molars were evaluated. According to the inclusion criteria, 38 teeth were excluded. The study sample comprised of 62 primary molars from 44 children with mean age of 85.806 ± 8.363 months. During the caries removal, in group II (TCR), 13 teeth were excluded from the sample due to pulp exposure, requiring pulpotomy. In group I (PCR), 24 teeth were treated and 17 teeth were clinically and radiographically followed up. In group II, 25 teeth were treated and 19 teeth were clinically and radiographically followed up (Flow Chart 1). The follow-up period was 4 to 6 months (mean = 5.028 ± 0.845 months).

All teeth of groups I (PCR) and II (TCR) showed clinical success at the 4- to 6-month follow-up period. No tooth exhibited pain, mobility, presence of fistula/abscess, and sensitivity to percussion. In group II, one tooth showed color alteration during the follow-up period.

**Figs 1A and B:**
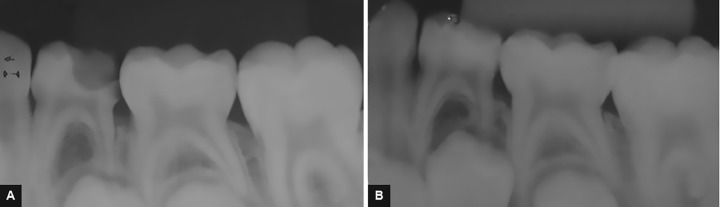
PCR on tooth #74. (A) Initial X-ray; (B) postoperative radiograph in the follow-up period

**Figs 2A and B:**
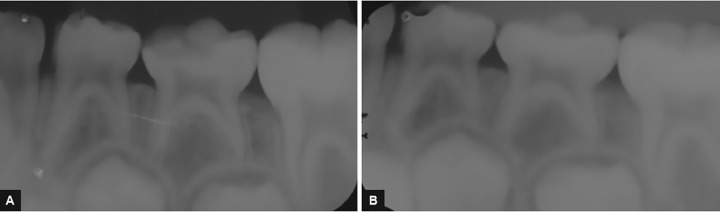
TCR on tooth #75. (A) Initial X-ray; (B) postoperative radiograph in the follow-up period

Radiographically, group I did not show internal and external resorption and periapical lesion (Fig. 1), but one tooth showed furcal lesion and advanced rhizolysis stage. In group II, external/internal resorption and periapical lesion were not observed (Fig. 2). One tooth showed furcal lesion, and another tooth showed advanced rhizolysis stage. At the radiographic assessment, the success rate of groups I and II was 94.2 and 89.6% respectively. In group I, one tooth (5.8%) showed two radiographic occurrences (furcal lesion and advanced rhizolysis stage), while in group II, one tooth showed furcal lesion (5.2%) and other tooth exhibited advanced rhizolysis stage (5.2%). The data of the radiographic assessments are described in Table 1. The comparison of the radiographic outcomes did not show statistically significant differences for any of the study criteria (p > 0.05).

**Flow Chart 1:**
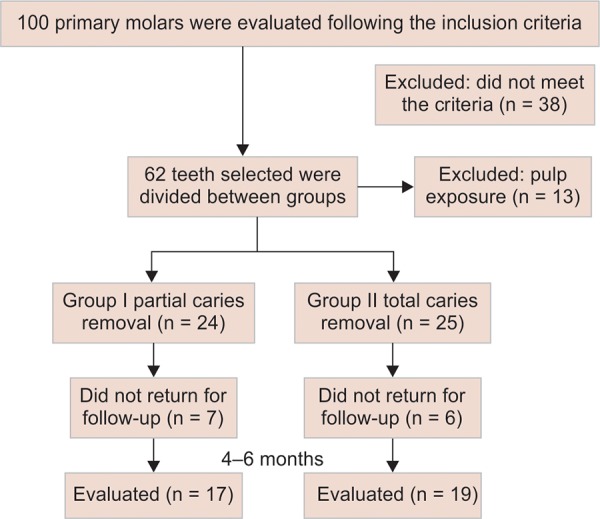
Fluxogram of the teeth treated (PCR or TCR)

**Table 1:** **Table 1:** Radiographic assessment—success (S) and failure (F) for PCR or TCR during the follow-up period

*Radiographic assessment (4-6 months)*																					
		*Internal resorption*				*External resorption*				*Furcal lesion*				*Periapical lesion*				*Advanced rhizolysis stage*			
*Groups*		*F*		*S*		*F*		*S*		*F*		*S*		*F*		*S*		*F*		*S*	
I (RP)		0		17 (100%)		0		17 (100%)		1 (5.8%)		16 (94.2%)		0		17 (100%)		1 (5.8%)		16 (94.2%)	
II (RT)		0		19 (100%)		0		19 (100%)		1 (5.2%)		18 (94.7%)		0		19 (100%)		1 (5.2%)		18 (94.7%)	

## DISCUSSION

Generally, the long-term prognosis of pulpal health may be more related to the pulp state during the initial treatment rather than to the technique used to remove the carious dentin.^18,19^ The premise of PCR technique is based on the modification of the microenvironment of the infected dentin under restoration.^5^ An advantage of PCR is the prevention of pulpal exposure during the removal of carious tissue, which avoids more complex treatments.^20^ Clinical, microbiological, and radiographic evidences have verified this technique’s success.^21,22^

During the removal of the carious tissue, the dentin consistency was taken into consideration. Carious dentin is divided into two different parts according to the morphological, biochemical, bacteriological, and physiological aspects: (1) The outer part is the infected dentin, irreversibly denatured, not capable of remineralization, which should be removed; (2) the inner, deepest part is the affected dentin, also contaminated, but reversibly denatured and capable of remineralization, which should be preserved.^23^ It is recognized that it is not easy to check how much tissue was removed.^6^ However, although somewhat subjective, the tactile sensation of reaching the firm dentin in pulp floor is the best guide.^6^ In this present study, PCR was performed according to standard methodology by Bressani et al,^13^ Franzon et al,^7^ and Dalpian et al.^5^ Where caries was selectively removed to leathery dentin,^7,9^ the PCR is a viable clinical approach because its outcomes did not statistically differ from those of TCR.^7,15^

The relationship between the clinical characteristic and bacterial colonization of carious dentin after PCR procedures was also studied.^15,24^ The PCR exhibited a greater number of microorganisms in the dentin; however, bacterial colonization was very similar to the total removal of caries in 3 to 6 months after restoration.^15^ The caries inactivating characteristics occur after sealing and restorative procedures;^24-26^ therefore, reopening the tooth to eliminate the remaining microorganisms is not required.^15^ All the procedures were performed in one stage and after PCR or TCR, the teeth were protected with calcium hydroxide cement.^15^ The cement of calcium hydroxide has been used in dentistry because of their good antimicrobial properties.^27,28^ In this present study, the calcium hydroxide cement was applied to the pulpal wall cavities of both groups.

The radiographic success was of 94.2 and 89.6% in groups I and II respectively, corroborating previous studies on conservative pulp therapy. Al-Zayer et al^29^ reported a success rate of 95% for indirect pulp capping after 12-month follow-up and suggested that this technique should be considered as an alternative for pulp therapy in teeth with deep carious lesions. Farooq et al^3^ and Vij et al^30^ found success rates for indirect pulp capping (94-93%) higher than those of pulpotomy with formocresol (74-70%) in the treatment of deep caries lesions. In a more recent study, Franzon et al^7^ reported high clinical and radiographic success rates (92% PCR, and 96% TCR) in the treatment of primary teeth with deep caries lesions after a 24-month follow-up. Dalpian et al^5^ also found high success rates for PCR (80.3%). However, similar to the studies of Farooq et al,^3^ Al-Zayer et al,^29^ and Vij et al,^30^ the study of Dalpian et al^5^ has a bias in the number of operators who performed the procedures, which did not happen in this study because only one previously trained and calibrated operator performed all procedures.

A growing trend for the treatment of caries is currently observed. Moreover, over the last years, the oral health of the population has improved, encouraging dental professionals to update for providing novel and better practices. This present study showed satisfactory clinical and radiographic outcomes for PCR procedure, suggesting that this minimally invasive approach might replace the TCR for primary teeth during a 4- to 6-month follow-up period. The study period range was the minimum needed to evaluate clinical and radio-graphic changes and was based on previous studies.^14,15^ No statistically significant differences were observed between PCR and TCR, corroborating the studies of Orhan et al,^31^ Lula et al,^15^ Phonghanyudh et al,^32^ and Franzon et al.^7^ Therefore, further studies are necessary to define protocols with more specific indications relating to the follow-up period, pulp capping material, and restorative materials. The comparison of the working time amount was not assessed in this present study so this could be considered for further studies. The PCR approach requires less operative time,^7^ a key feature for pediatric dentistry. Thus, this new strategy can be a great alternative for treating the most advanced stages of caries process, benefiting all patients.

## CONCLUSION

The PCR showed satisfactory clinical and radiographic outcomes, suggesting that this minimally invasive approach might replace the TCR when correctly indicated.
